# Quantitative trait locus *qLDC5* regulates primary root branching in an auxin-dependent manner

**DOI:** 10.1093/jxb/erag232

**Published:** 2026-05-27

**Authors:** Lam Thi Dinh, Bipin K Pandey, Yoshiaki Ueda, Nicola Leftley, Brian S Atkinson, Malcolm J Bennett, Matthias Wissuwa

**Affiliations:** Department of Applied Biology and Food Sciences, Faculty of Agriculture and Life Science, Hirosaki University, Hirosaki, Aomori 036-8561, Japan; School of Biosciences, University of Nottingham, Sutton Bonington LE12 5RD, UK; Crop, Livestock and Environment Division, Japan International Research Center for Agricultural Sciences (JIRCAS), Ohwashi 1-1, Tsukuba, Ibaraki 305-8686, Japan; School of Biosciences, University of Nottingham, Sutton Bonington LE12 5RD, UK; School of Biosciences, University of Nottingham, Sutton Bonington LE12 5RD, UK; School of Biosciences, University of Nottingham, Sutton Bonington LE12 5RD, UK; Institute of Crop Science and Resource Conservation (INRES), University of Bonn, Bonn 53115, Germany; University College Dublin, Ireland

**Keywords:** Auxin, lateral root density on crown root, primordia, *qLDC5*, rice

## Abstract

L-type lateral root (LLR) density determines root system architecture, affecting nutrient acquisition in rice (*Oryza sativa* L.), particularly under low-phosphorus conditions. Previous studies identified genotypic differences in LLR density and a quantitative trait locus (QTL) enhancing LLR density on crown roots (*qLDC5*). We showed that LLR densities on crown and primary roots were closely correlated and confirmed higher LLR density on primary roots in *qLDC5* donor DJ123 compared with the African variety NERICA4 using X-ray micro-computed tomography. We confirmed the *qLDC5* effect in a field experiment for LLR density on primary roots. LLR densities on primary and crown roots, therefore, appear under similar genetic control. Developmental analyses revealed that DJ123 and NDJ188—a derivative line harboring *qLDC5—*initiate more lateral root primordia than NERICA4, with a higher proportion progressing to elongation, but that exogenous auxin application reversed this ranking. Within *qLDC5*, auxin biosynthesis gene *OsYUCCA2* and auxin response factor *OsARF15* were up-regulated in DJ123. Transcriptome analysis revealed an indirect auxin-mediated regulatory network underlying LLR variation. Differentially expressed genes in DJ123 and NDJ188 were enriched for *ent*-kaurene and gibberellin metabolism, including the robust induction of *OsGA2ox5*. These findings suggest *qLDC5* increases lateral root density by coordinating gibberellin, auxin, and terpene pathways.

## Teaser

A rice genetic region was identified that increases lateral root density by coordinating hormone pathways, offering new insight into regulation of lateral root primordia initiation and elongation in a phosphorus-efficient genotype.

## Introduction

The primary root plays a crucial role in rice (*Oryza sativa* L.) seedling establishment, providing initial anchorage and absorbing water and nutrients essential for early growth. Lateral roots emerging from the primary root increase root system size, thereby expanding the soil volume explored for nutrients and water (Yamauchi *et al.*, 1987). As the rice plant develops, a large number (>100) of crown roots form at the basal stem nodes and these crown roots gradually take over functions the primary root provided during the early seedling stage (Gonzalez *et al.*, 2021). Lateral roots (LR) then emerge from crown roots and contribute prominently to nutrient (De Bauw *et al.*, 2020; Kuppe *et al.*, 2022) and water (Watanabe *et al.*, 2020) uptake. In rice, two types of LR are distinguished (Kawata and Shibayama, 1965): large L-type lateral roots (LLR) and small S-type lateral roots (SLR). LLRs branch further to develop SLRs and secondary LLRs, whereas SLRs are unbranched. LLRs can reach a maximum length of 10–15 cm with a diameter between 0.15 and 0.25 mm, whereas SLRs rarely exceed 1 cm in length with a diameter at or below 90 μm (Watanabe *et al.*, 2020; Wissuwa *et al.*, 2020; Gonzalez *et al.*, 2021).

The contribution of LRs to nutrient and water uptake is typically estimated from model simulations. Among root classes, SLRs contribute most to phosphorus (P) (Kuppe *et al.*, 2022) and water uptake (Watanabe *et al.*, 2020) under well-watered conditions, but in drying soil the relative importance of LLRs for P and water uptake increases, and this is more pronounced in a soil with low P availability (De Bauw *et al.*, 2020). Two quantitative factors are important in this regard: (i) the length of LRs and (ii) their branching density on the respective parent root (Gonzalez *et al.*, 2021; Kuppe *et al.*, 2022). A maximum LLR length of 146 mm has been reported in genotype DJ123 grown in an upland soil (Wissuwa *et al.*, 2020), but for rice grown in an anaerobic paddy soil, the maximum LLR length did not exceed 50 mm in a set of 12 cultivars from Bangladesh (Robin and Saha, 2015). These strong effects of the growth medium, together with the difficulty of clearly linking length to LLR root age at sampling, are likely responsible for the fact that very limited data on genotypic variation for LLR length are available. Genotypic variation in LLR branching density is better documented. Ranges reported are from 1.3 to 3.0 LLR cm^−1^ in upland rice parents ‘DJ123’ and ‘NERICA4’ used to develop a quantitative trait locus (QTL) mapping population (Dinh *et al.*, 2023) and from about 2.5 to 5.0 LLR cm^−1^ in a set of Egyptian lowland varieties (Hazman and Brown, 2018). In this latter set the LLR branching density (estimated using WhinRhizo) increased under drought stress to more than 20 LLR cm^−1^ in apical crown root sections sampled from deeper soil layers where water availability remained high. While these absolute numbers may be an overestimation of LLR density based on the un-supervised identification of root tips in WinRhizo, the clear increase in density highlights the importance of root plasticity in response to environmental stresses (Hazman and Brown, 2018).

Using the QTL mapping population derived from the Bangladeshi landrace DJ123 (high LLR density) and African upland rice variety NERICA4 (low LLR density), we recently identified a major QTL increasing L-type density on crown roots (*qLDC5*) (Dinh *et al.*, 2023). *qLDC5* explained 46% of the variation in LLR density in the mapping population, and 38% of the variation in P uptake (Dinh *et al.*, 2023). In this QTL mapping experiment, the focus was on lateral root branching on crown roots in older plants, rather than on the primary root, as the latter is often difficult to distinguish from older crown roots and can be severely damaged during excavations in the field. Crown roots offer the added advantage of being produced in large numbers, allowing for multiple measurements of lateral root density from a single excavated plant. In contrast, younger seedlings retain the primary root as the dominant structure. Assessing lateral root density on the primary root in these seedlings offers the benefit that the exact age of the root is known. Thus, controlling for developmental stage is possible, whereas the exact age of a crown root is typically not known, which adds a level of uncertainty when comparing LLR densities across crown roots. Since both methods have their advantages and disadvantages, clarifying to what extent lateral root densities on primary and crown roots are related and may be under similar genetic control is an important next step towards understanding underlying genetic and physiological factors.

Lateral root branching is known to be an auxin-dependent process (Péret *et al.*, 2009), but to what extent genotypic differences in LLR density can be attributed to auxin is not known. It is further unknown whether genotypic differences in LLR density are the result of differences in root primordia initiation or primordia elongation. These questions were addressed experimentally using the parental lines of the *qLDC5* mapping population and BC_2_ progeny lines segregating for the *qLDC5* locus.

The key objectives of the present study therefore are to (i) explore whether the *qLDC5* effect on increasing lateral root density is limited to crown roots or is also detectable on the primary root; (ii) examine whether the increase in lateral root density associated with *qLDC5* is primarily driven by enhanced formation of lateral root primordia or by more rapid elongation of formed primordia; (iii) identify candidate genes involved in the regulation of contrasting branching phenotypes in DJ123 and NERICA4; and (iv) investigate the effect of auxin and auxin-responsive gene expression on genotypic differences in LLR density.

## Materials and methods

### Plant materials and growing conditions

Plant materials used in this study are listed in Supplementary Table S1 according to each experiment. These include line NDJ188 selected from the DJ123×NERICA4 mapping population and backcrossed to NERICA4 to produce BC_1_F_6_ line AB199. Both NDJ188 and AB199 carry the positive DJ123 allele at *qLDC5*. A further backcross of AB199 to NERICA4 produced BC_2_F_3_ lines used for confirmation of the *qLDC5* effect. Seeds were sterilized with 70% ethanol for 30 s and rinsed with deionized water three times before being placed in a 28 °C incubator for 48 h for pre-germination.

### X-ray computed tomography imaging

Homogeneous pregerminated seeds were placed in 20 mm PVC tubing (‘soil columns’) with a 16 mm inner diameter and a height of 40 mm. Two columns were combined into a single working column with a total length of 80 mm. Approximately 90 g of loamy sand soil, with a bulk density of 1.2 g cm^−3^, was used to fully fill the column. Since there was not much lateral root development in the lower part of the column, it was removed prior to the scanning process to improve drainage. The seeds were grown in a rice growth chamber maintained at 30 °C, 12 h photoperiod, and 300 μmol m^−2^ s^−1^ light with 70% relative humidity. The root systems of 10-day-old rice seedlings were imaged using a Phoenix V|tome|X MDT 180 high resolution X-ray computed tomography (CT) system (Waygate Technologies, Wunstorf, Germany) at the Hounsfield Facility, University of Nottingham, UK. The scanning parameters were optimized to allow balance between a large field of view and a high resolution. Prior to CT scanning the soil columns were placed on a layer of drying paper for 1 d, to ensure the highest level of contrast between root and background. Each sample was then imaged using a voltage and current of 70 kV and 140 µA, respectively, at a voxel size resolution of 12 µm (giving a geometric magnification of 16.66 with a focus-to-image distance of 803.42 and focus-to-object distance of 48.205). The specimen stage rotated through 360 degrees at a rotation step increment of 0.15 degrees over a period of 40 min, so a total of 2400 projection images were obtained using the fastscan setting and multiscan of two sections with an exposure of 500 ms. Each scan was reconstructed using DatosRec software (Waygate Technologies). Radiographs were visually assessed for sample movement before being reconstructed in 16-bit depth merged volumes with a beam hardening correction of 8; no filtration was applied to the image. Reconstructed sections were processed in VG Studio MAX (version 2.2.0; Volume Graphics GmbH, Heidelberg, Germany).

Four plants per genotype were scanned. Depending on developmental stage, three to five 1 cm sections along the primary root of each plant were analysed to assess L-type density. In total, a minimum of 15 data points per genotype were used for statistical analysis.

### Greenhouse experiments

For the rhizobox experiment, five rice genotypes were grown in Plexiglas rhizoboxes of dimensions 30×30×2 cm (height×width×depth for inner dimensions) with three biological replicates. Soil properties and growing conditions followed Dinh *et al.* (2023). Plant roots were sampled at 35 days after sowing (DAS). For the pot experiment, 15 genotypes contrasting for the presence of *qLDC5* were grown in 1 liter Wagner pots filled with 900 g of unfertilized soil. Seedlings were grown in the greenhouse of Hirosaki University, Japan, for 2 weeks before sampling. Root scanning and phenotyping of lateral root traits were described in the previous study (Dinh *et al.*, 2023).

### Field experiment in Madagascar

Segregating F_3_ progeny lines derived from backcrossing line AB199 carrying the DJ123 allele at *qLDC5* with NERICA4 were grown in an upland field in Mahajanga, Madagascar, in 2025. The field was watered twice a week, and roots were sampled at 3 weeks after sowing. Roots of three plants per genotype per replication (with three replications) were excavated, gently washed, and then stored in zipped freezer bags with a small amount of water for further analysis. Roots were imaged by a Nikon D7200 digital SLR camera with a Sigma 18–250 1:3.5–6.3 DC Macro DS HSM lens, and L-type density was counted using ImageJ.

### Auxin treatments, lateral root primordia quantification, and RNA-seq

Pregerminated seeds were placed onto a mesh floating in trays containing 1.7 liters of deionized water. Seedlings were grown without external indole-3-acetic acid (IAA) or with a supply of 100 nM IAA applied at 3 DAS. Root tips of 2 cm were collected for RNA isolation after 1 h of IAA treatment. Up to 3 cm of root from the tip was sampled for lateral root primordia on the fifth day after IAA application. RNA samples were extracted using the RNeasy Plant Mini kit (Qiagen, Germany) and then subjected to next-generation sequencing to obtain 150 bp paired-end reads using the Illumina NovaSeq6000 sequencer. Raw data were additionally analysed using CLC Genomic Workbench (Ver. 22.0.2, Qiagen, Japan). The sequences with an estimated error probability higher than 5% (limit 0.05) were trimmed as low quality. Adapter was trimmed using automatic read-through adapter trimming. The trimmed reads were mapped to the Nipponbare reference transcript (IRGSP-1.0), and categorized and assigned to the transcripts using the EM estimation algorithm, and expression values for each gene were obtained by summing the transcript counts belonging to the gene. Transcripts per million values were then computed from the counts assigned to each transcript. A differential expression analysis was performed based on a negative binomial generalized linear model, which was used for fold change estimation as well. The whole transcriptome RNA-seq was normalized using the trimmed mean of *M*-values method.

Root sections were stained by incubating in a 0.2% solution of Basic Fuchsin dissolved in ClearSee solution, mixed 1:1 with an aqueous solution of Calcofluor White to stain cell walls. Roots were quickly rinsed twice and incubated in fresh ClearSee solution for at least 4 d to diminish autofluorescent compounds in root tissue. LLR primordia were then observed under a Leica SP8 confocal laser scanning microscope (Leica Microsystems, Wetzlar, Germany). Stained root samples were mounted in a drop of ClearSee and positioned on the Leica SP8 microscope. The excitation and emission wavelengths were set at 561 nm and 600–650 nm, respectively, for Basic Fuchsin, 405 nm and 425–475 nm, respectively, for Calcofluor White. LLR primordia were quantified in defined zones along the root.

Lateral root primordia were defined based on their morphological progression during lateral root development, which can be divided into distinct stages characterized by specific anatomical features and patterns of cell division (Malamy and Benfey, 1997). Elongated primordia were defined as late-stage lateral root primordia that had progressed to root emergence.

Root tips from primary roots images of auxin reporter line DR5:VENUS were analysed using ImageJ software. Fluorescence intensity within regions of interest was quantified as raw integrated density. Regions of interest were defined by drawing rectangular selections over the relevant areas of the captured images.

### Statistical analyses

Phenotypic data were analysed with the statistical software Statistix (Analytical Software, version 10.0) employing an ANOVA followed by appropriate post-hoc tests for significant factors (see figure legends for details). RNA-seq data were analysed using the CLC Genomic Workbench (Ver.22.0.2) after quality control testing and trimming of low-quality sequence (limit=0.05) and ambiguous nucleotides (max. two nucleotides allowed).

## Results

### L-type lateral root density on the primary and crown roots

**Fig. 1.**
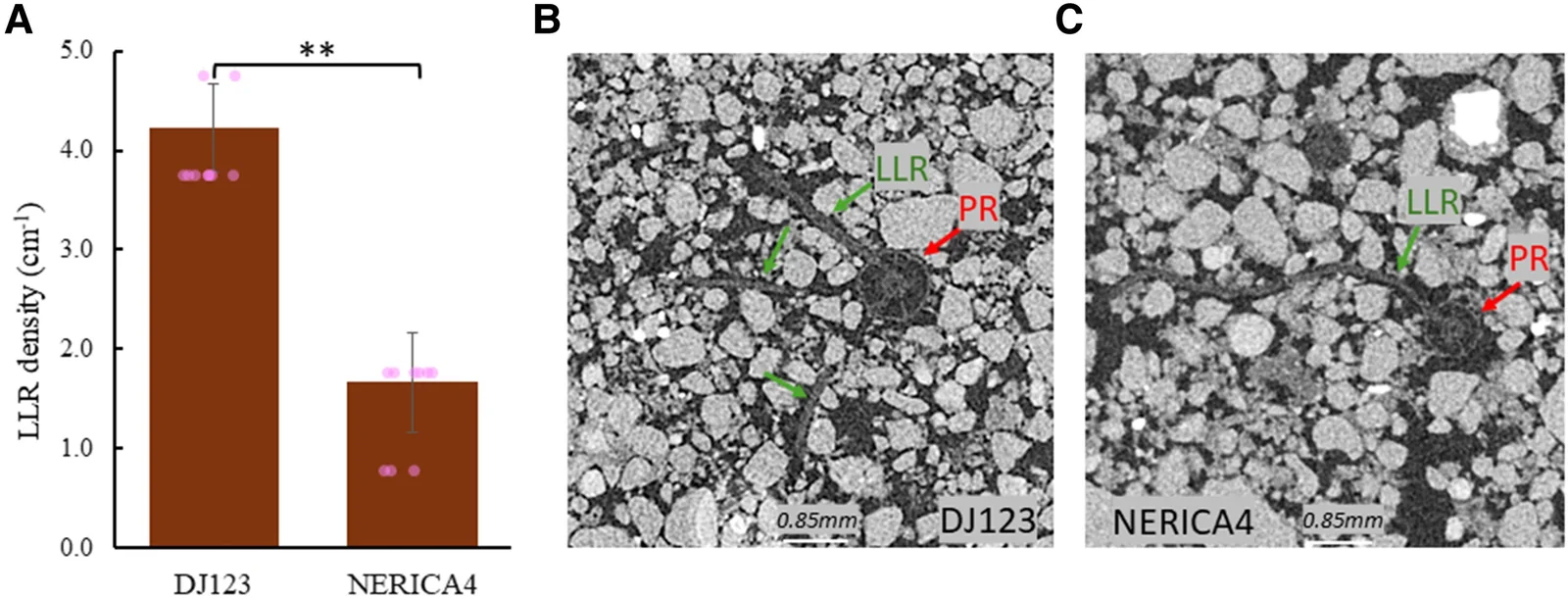
(A) L-type lateral root (LLR) density on the primary root (PR) of DJ123 and NERICA4, quantified using X-ray micro-computed tomography (CT) scanning. Values are means ±SD (*n*=5). Student’s *t*-test was performed, and significant difference is shown: **P<0.01. (B, C) Top-view X-ray micro-CT scanning images showing a higher number of LLRs emerging from the primary root of DJ123 (B) compared with NERICA4 (C). Plants were grown in soil and scanned at 10 d after germination.

Root excavation and removal from soil often risk damaging the root system (especially thin branches), making it difficult to accurately capture root architecture as it naturally occurs in soil. To confirm L-type lateral root (LLR) branching in parental genotypes without disturbing the roots, an experiment using X-ray micro-CT scanning was conducted. Results confirmed higher LLR density on the primary root in DJ123 with 4.2 LLRs cm^−1^ compared with 1.6 LLRs cm^−1^ in NERICA4 (Fig. 1).

**Fig. 2.**
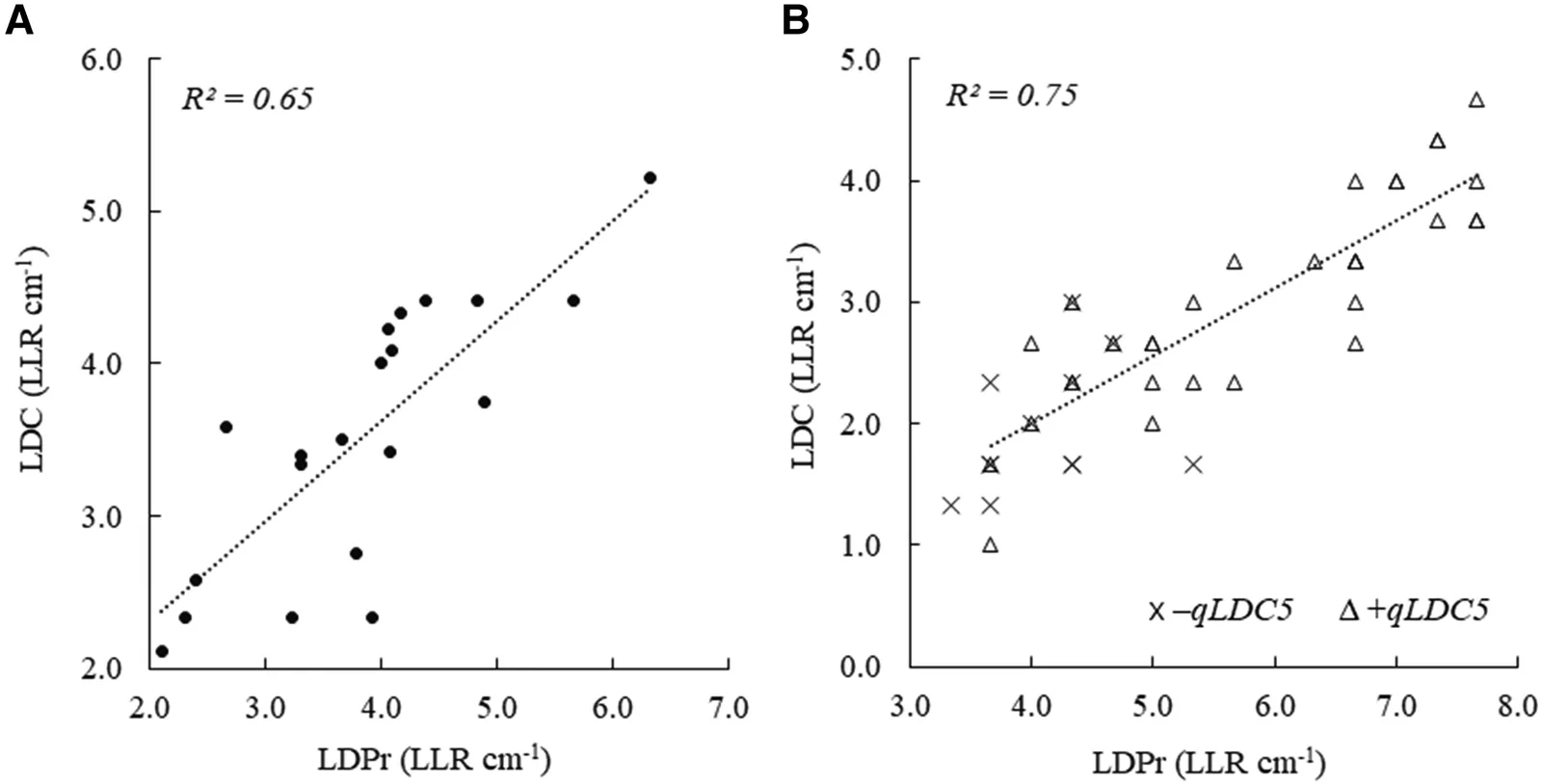
L-type lateral root densities on the primary root versus on crown roots. (A) Plants of five genotypes were grown in rhizoboxes and sampled 35 days after sowing (DAS). (B) Fifteen genotypes contrasting for the presence of *qLDC5* were grown in Wagner pots with 900 g of soil, with sampling at 15 DAS. Both experiments used an unfertilized Andosol. LDC, L-type density on the crown roots; LDPr, L-type density on the primary roots.

To determine the relation between L-type density on the primary (LDPr) and crown (LDC) roots, two experiments were conducted. In a rhizobox experiment with five genotypes, plants sampled at 35 DAS varied by 3-fold in LDPr (2.1–6.3 LLR cm^−1^) and more than 2-fold in LDC (2.1–5.2 LLR cm^−1^), and densities on both root types were closely correlated (*R*^2^=0.65) (Fig. 2A). An even tighter correlation (*R*^2^=0.76) between densities on both parent root types was detected in a pot experiment with 15 genotypes contrasting for the presence of *qLDC5* (Fig. 2B). The group carrying the positive *qLDC5* allele from DJ123 had 46% higher LLR density on the primary root compared with the −*qLDC5* group, and on crown roots the difference in density increased to 62%. LDPr varied 2-fold from 3.8–7.6 LLR cm^−1^ with almost 3-fold variation detected for LDC (1.4–4.2 LLR cm^−1^). In both experiments, average LLR density was 65–96% higher on the primary root compared with crown roots.

### Confirmation of the effect of *qLDC5* in a field experiment in Madagascar

**Fig. 3.**
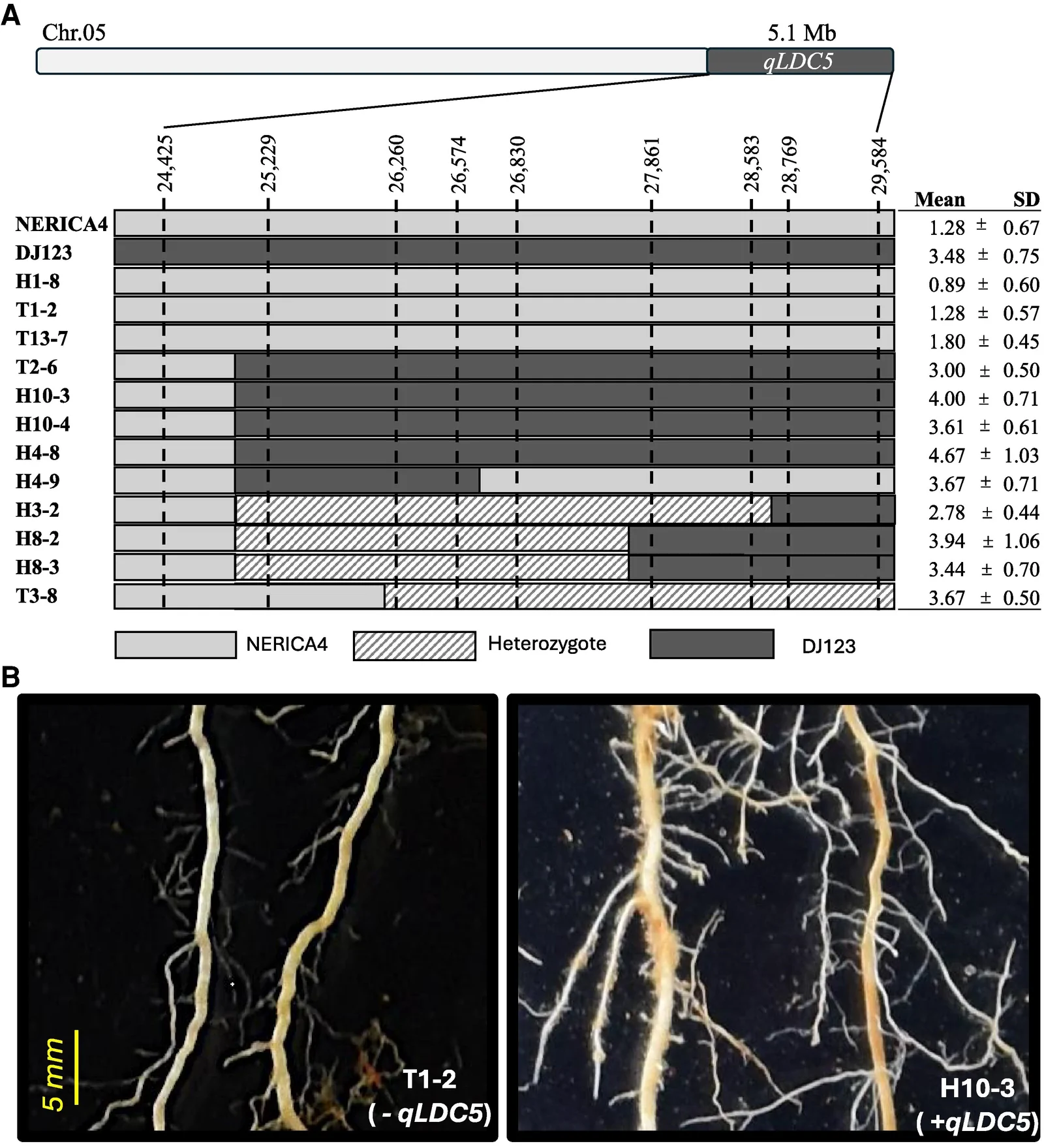
Evaluation of F_3_ progeny lines derived from backcrossing a line carrying the DJ123 allele at *qLDC5* with NERICA4, conducted in an upland field in Mahajanga, Madagascar, in 2025. (A) Graphical representation of genotypes on chromosome 5 of parents and 12 BC_2_F_3_ lines used to confirm the *qLDC5* effect on LLR branching density on primary roots. Marker positions on chromosome 5 (in kb) are indicated for the region with a DJ123 introgression from 24 425 kb until the end of chromosome 5. (B) Root photos of presentative lines excavated from the field. Line T1-2 was a negative control while Line H10-3 carried the positive allele at *qLDC5*.

The above branching data indicate that LLR densities on crown and primary roots are highly related, and with slightly higher variability on the primary root, phenotyping for LDPr should be equally or better suited for further fine-mapping of *qLDC5*. We therefore determined LLR density on the primary root of segregating F_3_ progeny lines derived from backcrossing a line carrying the DJ123 allele at *qLDC5* with NERICA4 in a first attempt to confirm the effect of *qLDC5*. Lines were grown in a low-input field in Madagascar and plants were excavated 3 weeks after sowing. Recurrent parent NERICA4 had an average LDPr of 1.3 LLR cm^−1^, and the three progeny lines carrying the NERICA4 allele at *qLDC5* ranged from 0.9 to 1.8 LLR cm^−1^ (Fig. 3). In contrast, lines with the DJ123 allele ranged from 3.0 to 4.7 LLR cm^−1^. Heterozygous lines varied considerably, ranging from 2.8 to 3.9 LLR cm^−1^.

### Does lateral root density depend on primordia initiation or subsequent elongation?

**Fig. 4.**
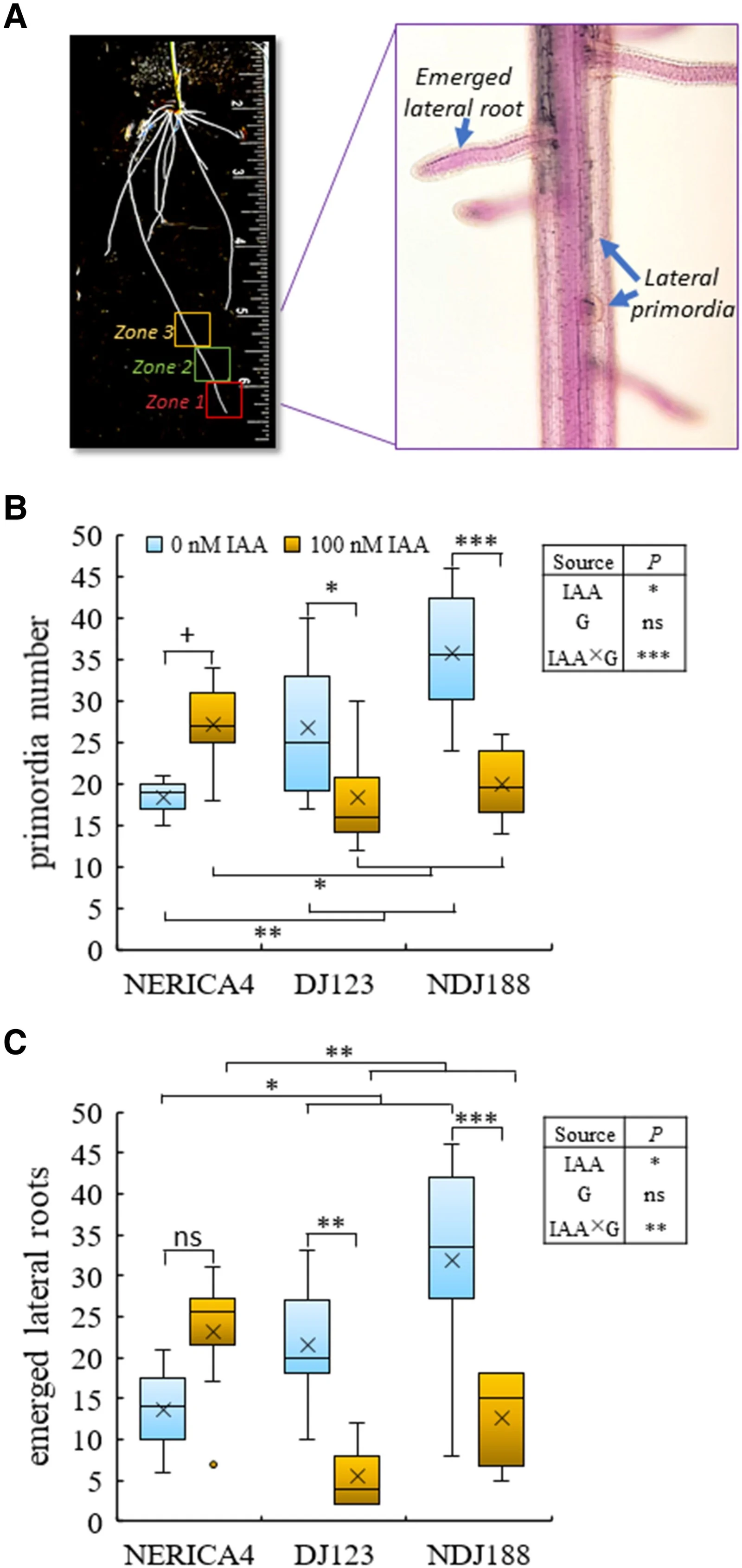
Lateral root primordia initiation and elongation in the primary root tip region of parental genotypes DJ123, NERICA4, and derived line NDJ188. (A) Most primordia were already elongated into lateral roots, and non-elongated primordia were counted in three zones corresponding to the first, second, and third centimeter from the root tip. (B, C) The total number of lateral root primordia (initiated and elongated) (B) and number of emerged lateral roots (C) are shown for 7-day-old primary roots. Plants were grown without external indole-3-acetic acid (IAA) or with a supply of 100 nM IAA applied 3 d after germination, with sampling done on the fifth day after IAA application. Data shown are for combined zones 2 and 3, as the lateral roots had generally not yet emerged in zone 1. The two-way ANOVA detected highly significant IAA×genotype (G) interactions. A set of five orthogonal contrasts was then employed to test the most relevant differences (treatment effects within genotypes (3) and differences between NERICA4 and the two carriers of *qLDC5* at each IAA treatment (2)). Significant differences between means are shown: ^+^*P*<0.10, *P<0.05, **P<0.01, ***P<0.001; ns, non-significant difference.

We have shown that L-type lateral root density on crown root and primary root is closely related in parental lines and that differences in LDPr correspond to allelic differences at *qLDC5*. Lateral root primordia begin to form approximately 1.5 mm from the root tip (Rebouillat *et al.*, 2009), and emerge 3 cm from the tip. Within the second and third centimeter from the tip (zone 2 and 3; Fig. 4A), DJ123 and NDJ188 had initiated on average 27 and 37 primordia, respectively, and that was significantly higher compared with NERICA4 with only 18 primordia (Fig. 4B). Of the total initiated primordia, a majority had already elongated into lateral roots and this was especially pronounced in NDJ188 (of 37 primordia 32 had elongated into lateral roots; Fig. 4C). Importantly, L-type primordia were rarely detected in most cases (Supplementary Fig. S1).

Moreover, genotypes reacted differently to the application of external auxin. Addition of 100 nM IAA to the growth medium increased primordia number in NERICA4 by 48% but the opposite effect was seen in DJ123 and NDJ188 (Fig. 4B, C). The external IAA addition had an even stronger positive effect on the number of elongated primordia in NERICA4 (+70%), leading to a similar number of elongated lateral roots compared with DJ123 and NDJ188 without IAA addition. In contrast, the sensitivity to external IAA increased for elongated lateral root number in DJ123 and NDJ188, causing a reduction of 74% and 60%, respectively, in these genotypes. Taken together, these data suggested that allelic variation at *qLDC5* influences lateral root density through effects on both primordia initiation and their subsequent elongation, with external auxin revealing pronounced genotypic differences in auxin responsiveness.

### Auxin signaling enhancement after external IAA application increases under phosphorus deficiency

**Fig. 5.**
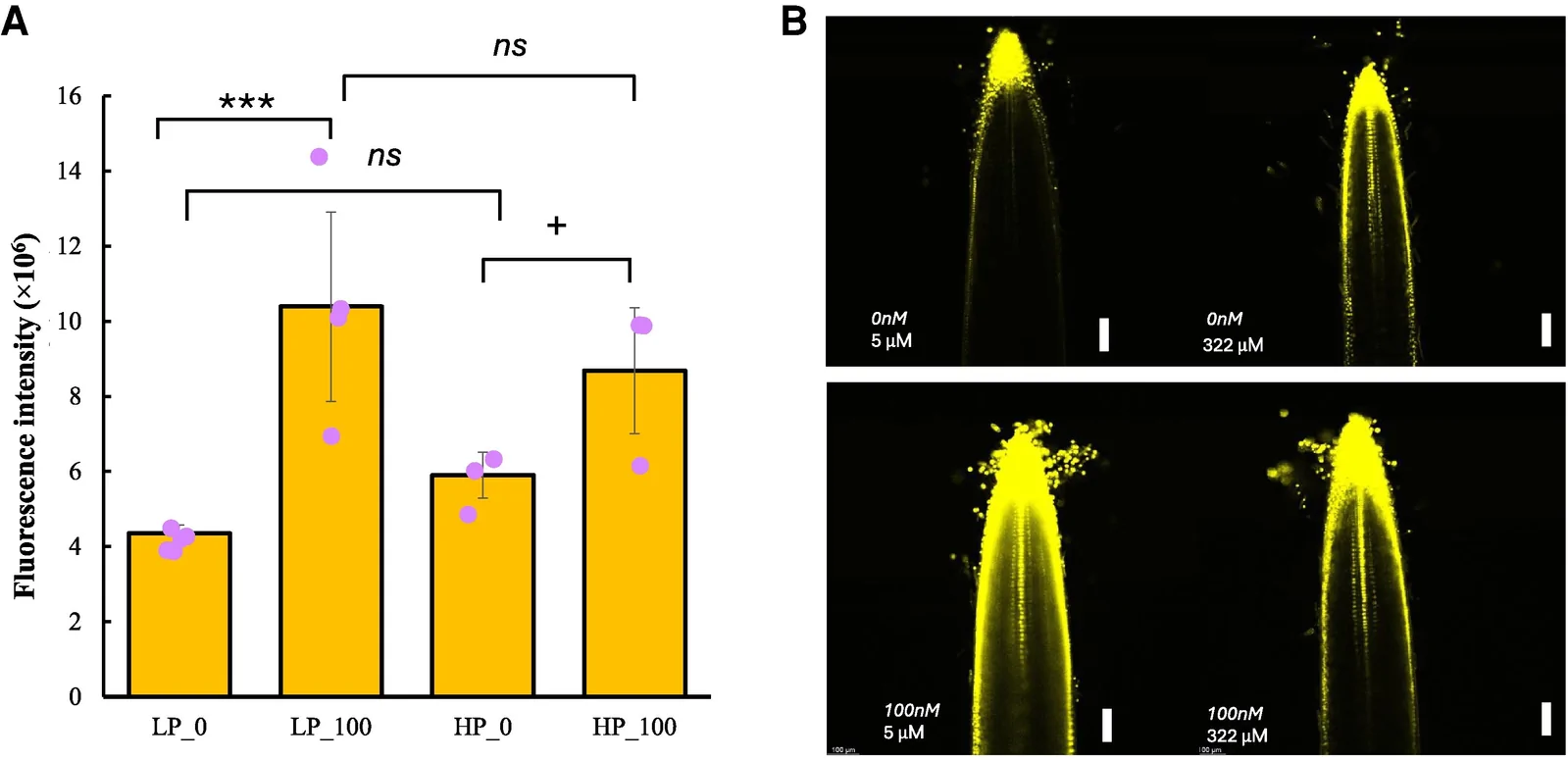
Expression of the auxin response reporter DR5:VENUS in a Dongjin genetic background. Plants were grown in a nutrient solution to which phosphorus (P) was added either at a low (LP, 5 μM) or high (HP, 322 μM) concentration. Indole-3-acetic acid (IAA) was added at a concentration of 100 nM (HP_100 and LP_100), and a control with 0 nM IAA (HP_0 and LP_0). An ANOVA indicated highly significant IAA effects (*P*=0.0003) that were further tested using orthogonal contrasts. Significant differences between means are shown: ^+^*P*<0.10, ***P<0.001; ns, non-significant differences. Bar: 100 μm.

Identification of *qLDC5* was originally done in soils characterized by very low P availability, and the focus on low-P soils was continued in the present study with similar low-P Andosols used in pot experiments (Fig. 2) and a highly P-fixing unfertilized Oxisol used to confirm the effect of *qLDC5* in Madagascar (Fig. 3). Given this context, it is therefore important to determine the extent to which P deficiency can affect auxin signaling. The auxin reporter line DR5:VENUS in a Dongjin background (Huang *et al.*, 2022) was used in a two-factorial experiment with P supply and IAA addition as factors. External IAA application significantly increased the DR5:VENUS signal in the root tip region in both high-P and low-P treatments (Fig. 5). Interestingly, the signal increase was more pronounced in the low-P treatment, where external IAA almost tripled signal intensity (from 4.3×10^6^ to 11.5×10^6^) compared with a 60% increase (from 5.9×10^6^ to 9.8×10^6^) under high-P conditions. This enhanced auxin responsiveness under P deficiency is consistent with the notion that low P conditions sensitize the root system to auxin signaling, thereby promoting lateral root development (Péret *et al.*, 2014; Giri *et al.*, 2018).

### Transcriptome analysis reveals an indirect auxin-mediated regulatory pathway underlying genetic variation in root development, with the involvement of jasmonate and gibberellin metabolism

**Fig. 6.**
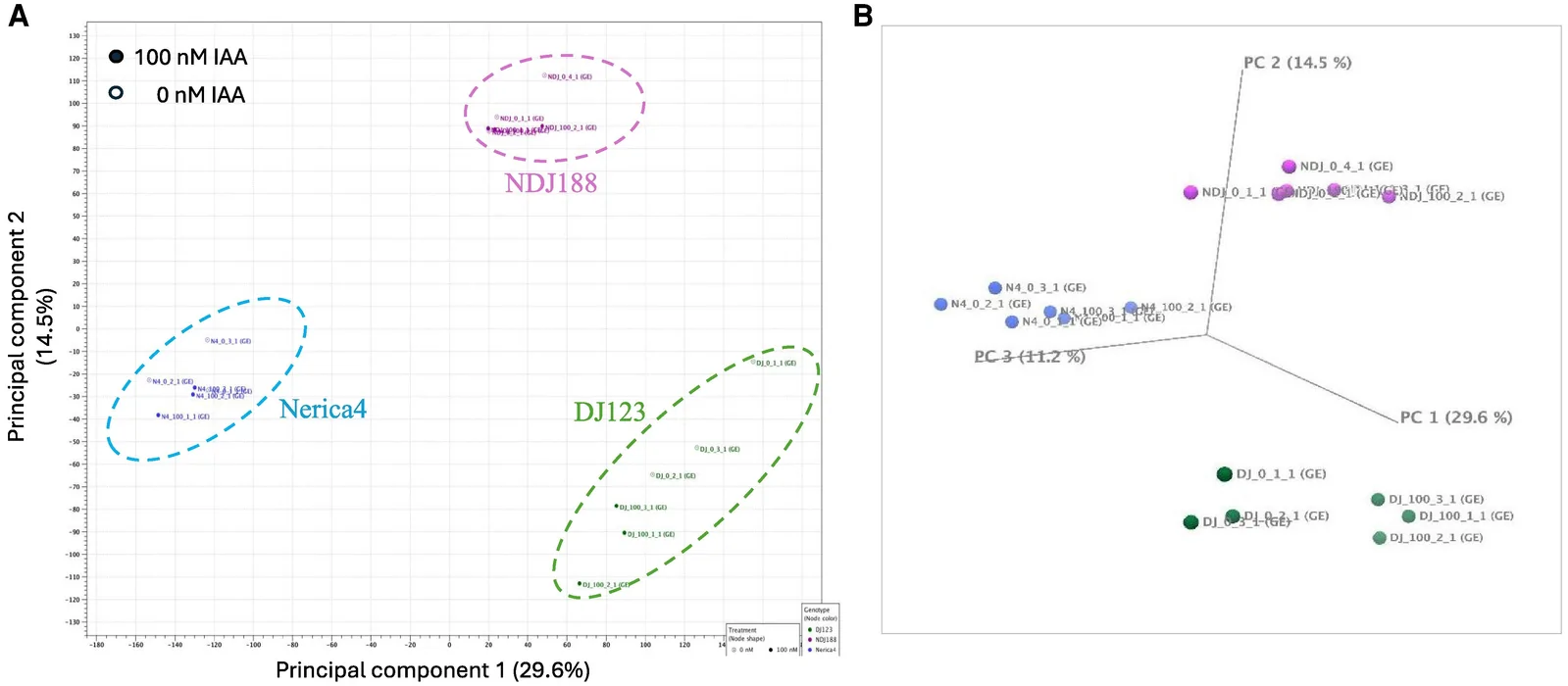
Principal component scatter plot of RNA-seq data of three genotypes, DJ123, NDJ188 and NERICA4 at two indole-3-acetic acid (IAA) treatments: 100 nM (closed dots) and 0 nM (open dots) in two-dimensional view (A) and three-dimensional view (B). Three replications for each treatment and genotype were analysed. The PCA analysis was performed in CLC Genomic Workbench (Ver.22.0.2) after quality control testing and trimming of low-quality sequence (limit=0.05) and ambiguous nucleotides (max. two nucleotides allowed).

The transcriptome analysis of 3 cm root tip samples from control and IAA treated roots indicated genotypes were the dominant source of transcriptional variation, accounting for 29.6% of the total variance represented by principal component (PC) 1 (Fig. 6). The auxin response was most pronounced in DJ123 and captured by PC3 accounting for 11.2% of the variation. PC2 separated NDJ188 from both parental genotypes.

**Fig. 7.**
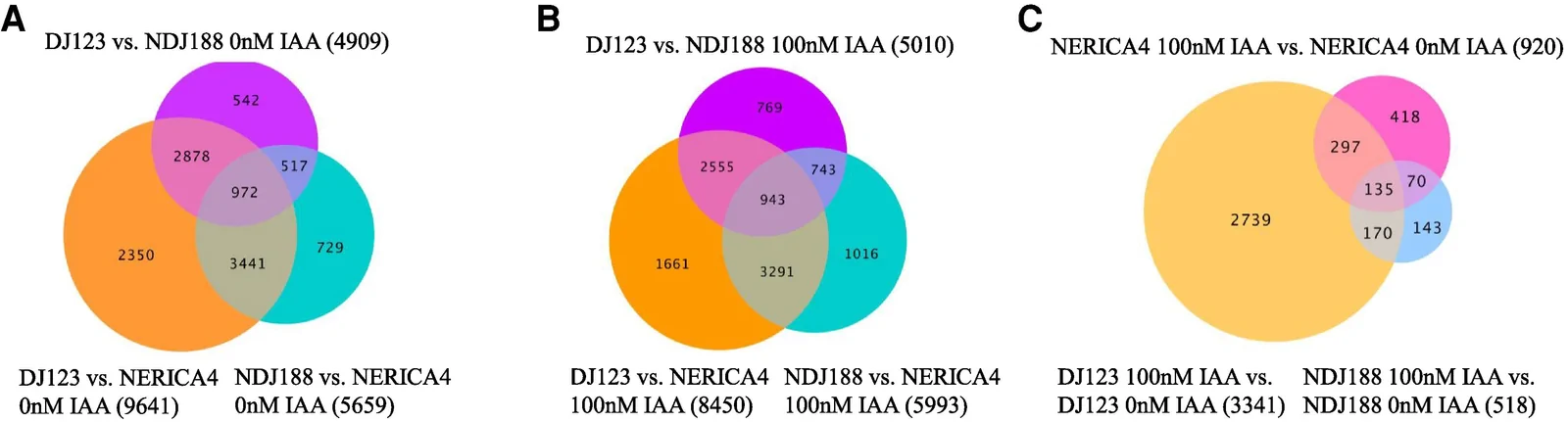
Venn diagram of differentially expressed genes (DEGs) for genotype comparisons at the 0 IAA (A) and 100 nM IAA treatments (B), and DEGs in response to IAA addition within genotypes (C). The Venn diagrams are shown based on minimum absolute fold change of 1.5 and a false discovery rate *P*-value <0.05. The number of DEGs for each comparison is shown in parentheses.

In the non-IAA control, 9641 differentially expressed genes (DEGs) were detected between DJ123 and NERICA4 compared with 5659 DEGs between NDJ188 and NERICA4 and 4909 DEGs between DJ123 and NDJ188 (Fig. 7A). In the IAA treatment, the number of DEGs decreased to 8450 for the DJ123 vs. NERICA4 comparison but increased marginally for the comparisons including NDJ188 (Fig. 7B). Within genotypes, the IAA treatment had a stronger effect in DJ123 compared with NERICA4 and NDJ188, revealing a high transcriptomic sensitivity to exogenous IAA in DJ123 (Fig. 7C). Given that DJ123 and NDJ188 exhibited similar branching pattern to exogenous IAA, we focused on DEGs shared between DJ123 and NDJ188 relative to NERICA4. The number of shared DEGs decreased only slightly (from 3441 under 0 nM IAA to 3291 under 100 nM IAA treatment. Notably, only 170 DEGs were specifically responsive to IAA in both DJ123 and NDJ188 but not affected by IAA in NERICA4 (Fig. 7C).

**Fig. 8.**
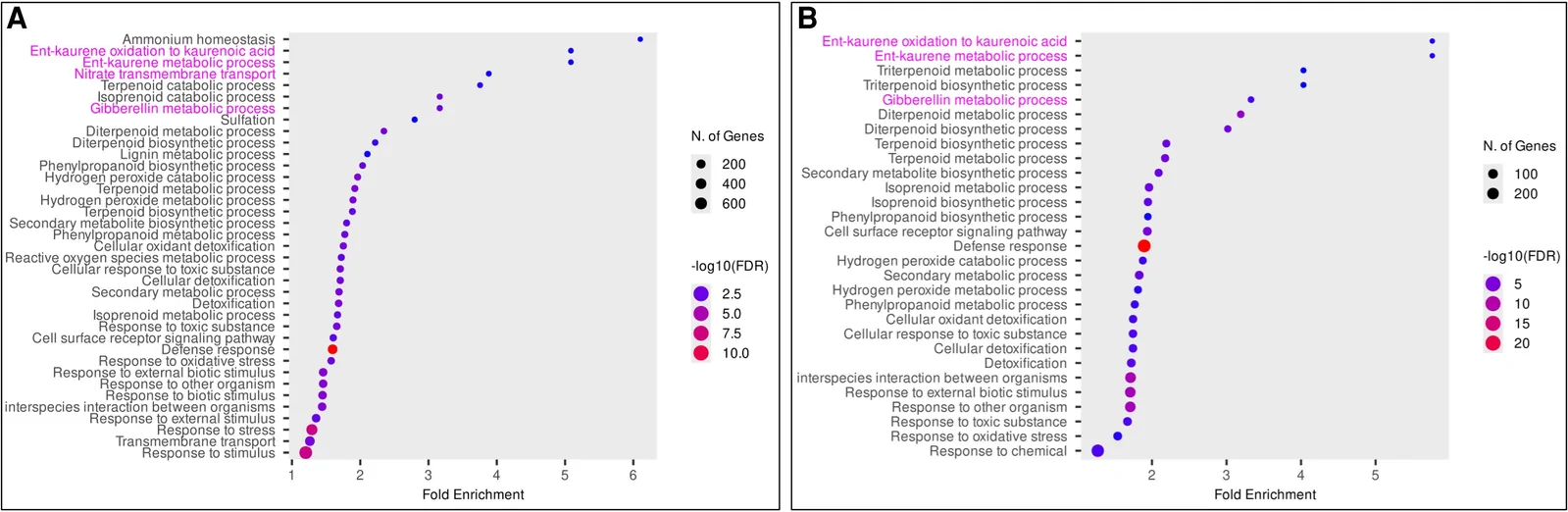
Gene enrichment analysis of differentially expressed genes (DEGs) in DJ123 versus NERICA4 at 0 nM indole-3-acetic acid (IAA) (A) and 100 nM IAA treatments (B). Dot-plots showing most significantly enriched Gene Ontology biological processes among DEGs (log_2_ fold change>1, *P*<0.05). Enriched categories include pathways related to gibberellin (GA) metabolism and nitrate transport (A). Notably, the same GA-related pathways are also enriched in DJ123 roots following 100 nM auxin (IAA) treatment compared with NERICA4 (B). GA-associated terms and nitrate transmembrane transport are highlighted in pink. FDR, false discovery rate.

**Fig. 9.**
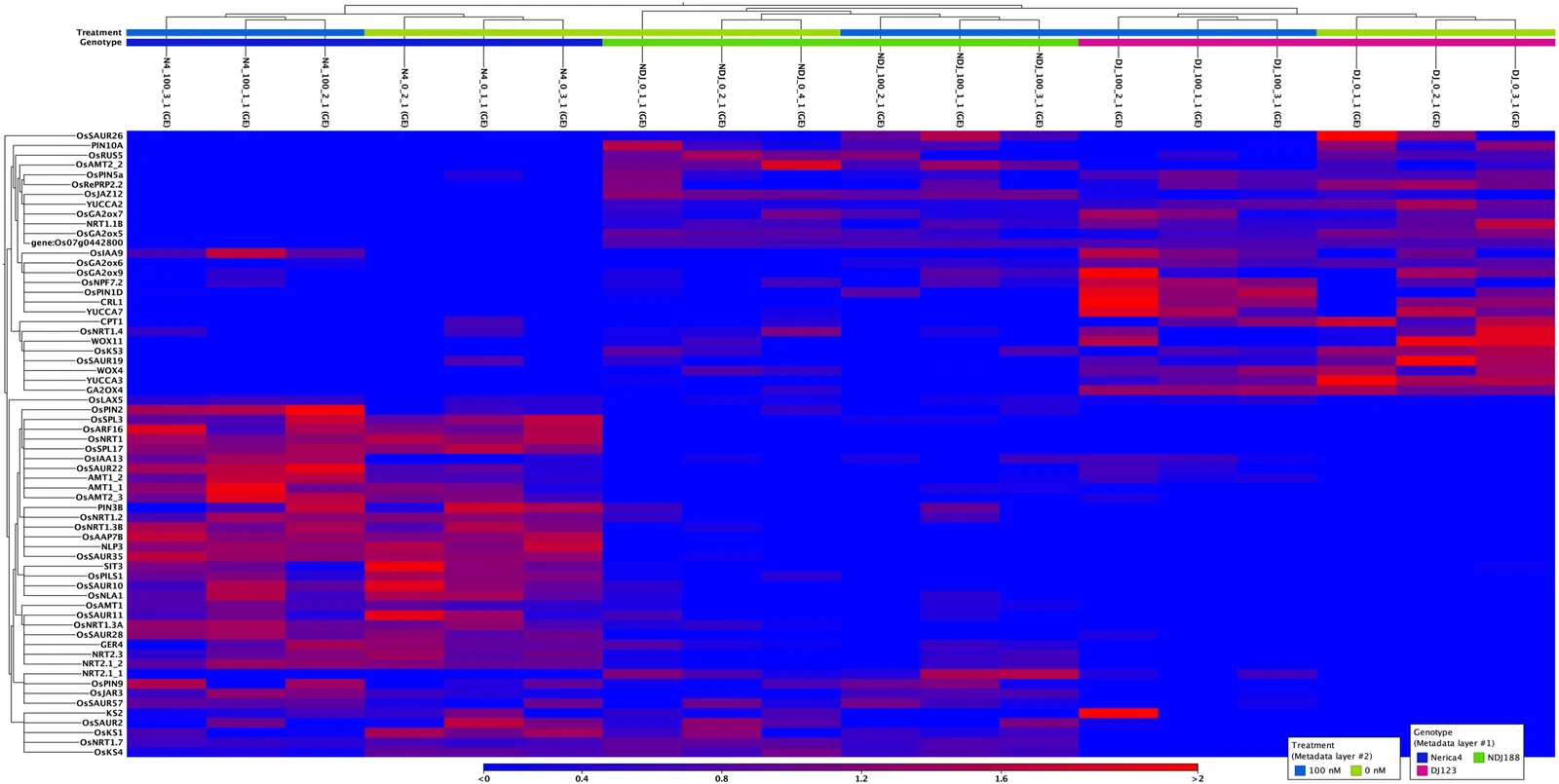
Heat Map for RNA-Seq of 64 differentially expressed genes (DEGs) selected from 9641 DEGs significantly expressed in DJ123 compared with NERICA4. The DEGs were selected based on their annotation contribution to root development (*CRL1*, *GER4*, *OsLAX5*, *OsRePRP2.2*, *WOX11*, *WOX4*, *gene:Os07g0442800* (*OsJAUP1*), *OsRUS5*, *OsSPL3*, *OsSPL17*), auxin biosynthesis and signaling (*CPT1*, *OsIAA13*, *OsIAA9*, *OsJAR3*, *OsJAZ12*, *OsPIN1D*, *OsPIN2*, *OsPIN5a*, *OsPIN9*, *OsSAUR11*, *OsSAUR19*, *OsSAUR2*, *OsSAUR26*, *OsSAUR28*, *OsSAUR57*, *PIN3B*, *YUCCA2*, *YUCCA3*, *YUCCA7*, *OsARF16*, *PIN10A*, *OsSAUR10*, *OsSAUR22*, *OsSAUR35*, *OsPILS1*), *ent*-kaurene synthase (*KS2*, *OsKS1*, *OsKS3*, *OsKS4*), gibberellin oxidase (*GA2OX4*, *OsGA2ox5*, *OsGA2ox6*, *OsGA2ox9*, *OsGA2ox7*), nitrogen transporter and nitrogen utilization efficiency (*NRT1.1B*, *OsNRT1.3B*, *OsNPF7.2*, *OsNRT1*, *NRT2.1_1*, *NRT2.1_2*, *NRT2.3*, *OsNLA1*, *OsNRT1.2*, *OsNRT1.3A*, *OsNRT1.4*, *OsNRT1.7*, *NLP3*, *OsAMT2;2*, *OsAMT1*, *AMT1;1*, *AMT1;2*, *OsAAP7B*, *OsAMT2;3*), and jasmonic acid-mediated terpenoid biosynthesis (*SIT3*). Each column corresponds to one sample, and each row corresponds to a gene. The samples are clustered into three groups according to three genotypes DJ123, NDJ188, and NERICA4. The red and blue colors reflect the expression level. The expression values are normalized log counts per million values.

Gene Ontology (GO) analysis of DEGs from the DJ123 vs. NERICA4 comparison revealed a strong enrichment of genes mainly involved in three processes (Fig. 8): (i) gibberellin (GA) metabolism, where *ent*-kaurene oxidation represents a biosynthetic step leading to GA production; (ii) triterpenoid metabolism that can affect auxin transport via the localization of PIN transporters; and (iii) ammonium vs. nitrate assimilation (in the 0 IAA control only). Interestingly, *OsJAUP1* and *OsGA2ox5* were extremely and specifically up-regulated in DJ123 compared with NERICA4, with the log_2_ fold change being 14.39 and 10.97, respectively (Fig. 9; Supplementary Table S2).

This enrichment also suggests that DJ123 may preferentially engage nitrate-related pathways under baseline conditions by highly expressed *NRT1.18* and *NPF7.2* genes, whereas NERICA4 may rely more heavily on ammonium-based nitrogen metabolism by highly expressed *AMT1_1/2* and *AMT2_3* genes (Fig. 9). Such differences are particularly relevant in the context of P limitation, as preferential nitrate uptake was shown to positively correlate with internal P utilization efficiency and P uptake efficiency in rice (Ueda and Wissuwa, 2022). Enhanced nitrate assimilation in DJ123 may therefore contribute to improved P acquisition and utilization under low-P conditions, potentially reinforcing root architectural traits such as increased lateral root density and elongation.

On the other hand, among the 170 IAA-affected DEGs, genes encoding WUSCHEL-related homeobox transcription factor (*OsWOX10*), which is involved in meristem activity and lateral root primordia development, and Auxin Response Protein 1 (*ARP1*) were up-regulated in both DJ123 and NDJ188 (Supplementary Fig. S2). We further focused on genes located within the *qLDC5* region, which contained five auxin-related genes (Supplementary Fig. S3). *OsYUCCA2* and *OsARF15* showed >10-fold higher expression in DJ123 relative to NERICA4. Notably, even after auxin treatment, their expression remained substantially elevated in DJ123 (>5-fold higher than NERICA4). These results suggest that the enhanced induction of *OsYUCCA2* and *OsARF15* is likely a key factor contributing to the superior branching capacity of DJ123 compared with NERICA4.

## Discussion

### L-type lateral root densities on primary and crown roots are closely related and controlled by *qLDC5*

A key objective of this study was to determine whether genotypic differences in L-type lateral root density are consistent on crown roots and the primary root, thereby allowing phenotypic evaluation of either root class. Two independent sets of genotypes were compared and LLR densities on both root classes were closely related, ranging from an *R*^2^ of 0.65 in a set of four genotypes to an *R*^2^ of 0.76 in near-isogenic lines derived from a *qLDC5* fine-mapping population (Fig. 2). These consistent correlations indicate that genotypic effects on LLR density are largely conserved across parent root types. Despite this consistency, absolute LLR density was substantially higher on the primary root, which exhibited 65–96% greater LLR density compared with crown roots. This systematic difference suggests that while the developmental regulation of LLR formation is genotype dependent and shared across root classes, the magnitude of lateral root production is also influenced by root type-specific factors.

Given this apparent close relation between LLR densities on both parent root types, we used LLR density on the primary root in an attempt to confirm the effect of *qLDC5* in a field experiment conducted in a target environment in Madagascar. Lines carrying the NERICA4 allele at *qLDC5* had a LDPr of 1.32 roots cm^−1^ compared with 3.79 for carriers of the DJ123 allele, and this represents the first confirmation of the *qLDC5* effect. Based on the segregation pattern, one may conclude that the *qLDC5* locus is flanked by markers at 25 229 kb and 27 861 kb on chromosome 5 (Fig 3). This 2.632 Mb interval contains 476 annotated genes (IRGSP-1.0_representative_annotation) and therefore remains too large to positionally identify candidate genes at this point. Further fine-mapping with remnant heterozygous lines such as H3-2, H8-2, H8-3, or T3-8 is therefore needed to increase the precision of mapping to a level where positional cloning of the underlying gene is possible.

Given that lateral root densities on the crown and primary root are strongly correlated, either trait can be used to evaluate LLR density and identify genetic factors underlying *qLDC5*. LDPr also offers the advantage that the developmental age of the primary root is known and uniform across seedlings germinating at the same time, as it emerges at germination, enabling standardized measurements at clearly defined time points. In contrast, crown root sampling requires selecting one or more crown roots, each possibly having a slightly different age. This can introduce developmental noise into the phenotype data, which may be amplified considering that crown roots may differ in root elongation rate (Takahashi and Pradal, 2021). A further advantage of using LDPr is the higher overall density on primary root. In contrast, low-branching genotypes of crown roots often yield very low values (e.g. 1 or 2 roots cm^−1^), which can skew the data and complicate analyses. These biological and methodological considerations support the use of primary root-based LLR density (LDPr) for confirming the *qLDC5* effect in segregating progeny derived from the DJ123×NERICA4 backcross, suggest that primary root phenotyping may be equally or better suited than crown root phenotyping for future fine-mapping and validation efforts.

### Challenges in interpreting lateral root primordia identity during early development

The current dataset in Fig. 4 captured lateral root primordia without distinction between SLR and LLR, and although an attempt was made to classify these in the original analysis, few L-type primordia could clearly be distinguished within the first 3 cm of root tip tissue (Supplementary Fig. S1). This may reflect a true density of LLR primordia, but it could also result from sampling roots at a very early developmental stage; L-type primordia could be morphologically distinguishable as zones 2–3 mature further. Previous studies in rice have shown that S- and L-type lateral roots differ in their developmental timing, size, and emergence dynamics, suggesting that L-type primordia may not be readily distinguishable at early stages (Inukai *et al.*, 2001, 2005). Several explanations are therefore possible: (i) LLR primordia are initiated later than S-type primordia, such that S-type primordia appear first, followed by L-type primordia at later time points, as proposed for rice lateral root development by Inukai and colleagues (Inukai *et al.*, 2001). (ii) LLR primordia are initiated at very early stages but are not detectable without higher-resolution approaches (e.g. microtome sections or gene expression reporter analysis). It is also possible that L-type primordia are larger and therefore develop more slowly, delaying their morphological distinction. And (iii) At these early developmental stages, S- and L-type primordia cannot be reliably distinguished. In this case, one could assume that a certain proportion of the observed primordia will ultimately become L-type, based on established S/L ratios. For example, if 30 primordia are detected, approximately 10–20% might be expected to develop into L-type primordia. Nevertheless, quantifying emerged lateral roots would provide a convenient phenotypic readout, but it cannot disentangle potential genetic differences between primordium initiation and elongation.

### The effect of auxin on lateral root primordia initiation and elongation and transcriptomic evidence on the involvement of auxin signaling

Branching is a key developmental trait predominantly regulated by auxin, which accumulates at pre-branch sites in xylem pole pericycle cells (Dolan *et al.*, 1993; Péret *et al.*, 2009). An auxin biosynthesis gene, *OsYUCCA2*, located within the *qLDC5* region was expressed in DJ123 but not in NERICA4, regardless of IAA treatment, and expression in DJ123 was reduced in the IAA treatment (Fig. 9). In addition, the auxin response factor *OsARF15* is also located within *qLDC5* with >2-fold higher expression in DJ123 compared with NERICA4 under control conditions. This difference decreased with addition of IAA. These differential expression patterns likely lead to higher auxin levels in DJ123 and may explain its sensitivity to external auxin application. However, confirmation of whether *OsYUCCA2* or *OsARF15* underlies the *qLDC5* locus will require further fine-mapping.

Beyond auxin-related pathways, GA-associated pathways remained among the most highly enriched categories in DJ123 even after auxin treatment (Fig. 8), indicating that GA biosynthesis in DJ123 operates largely independently of auxin-induced transcriptional changes. Single-cell transcriptomic analyses have shown that such hormonal pathways can be differentially coordinated across root tissues under environmental and hormonal perturbations (Zhu *et al.*, 2025). Notably, *Jasmonate Upregulated Protein 1* (*OsJAUP1*), which has been reported to regulate auxin-dependent root development through expression in the root cap and epidermal cells that protect meristematic stem cells and emerging lateral roots (Muzaffar *et al.*, 2024), was expressed at extremely high levels in DJ123 and NDJ188 but was down-regulated in NERICA4. The strong expression of *OsJAUP1* in DJ123 and NDJ188 further supports a role for jasmonate–auxin interactions in shaping lateral root development, potentially through regulation of meristem protection and lateral root emergence.

**Fig. 10.**
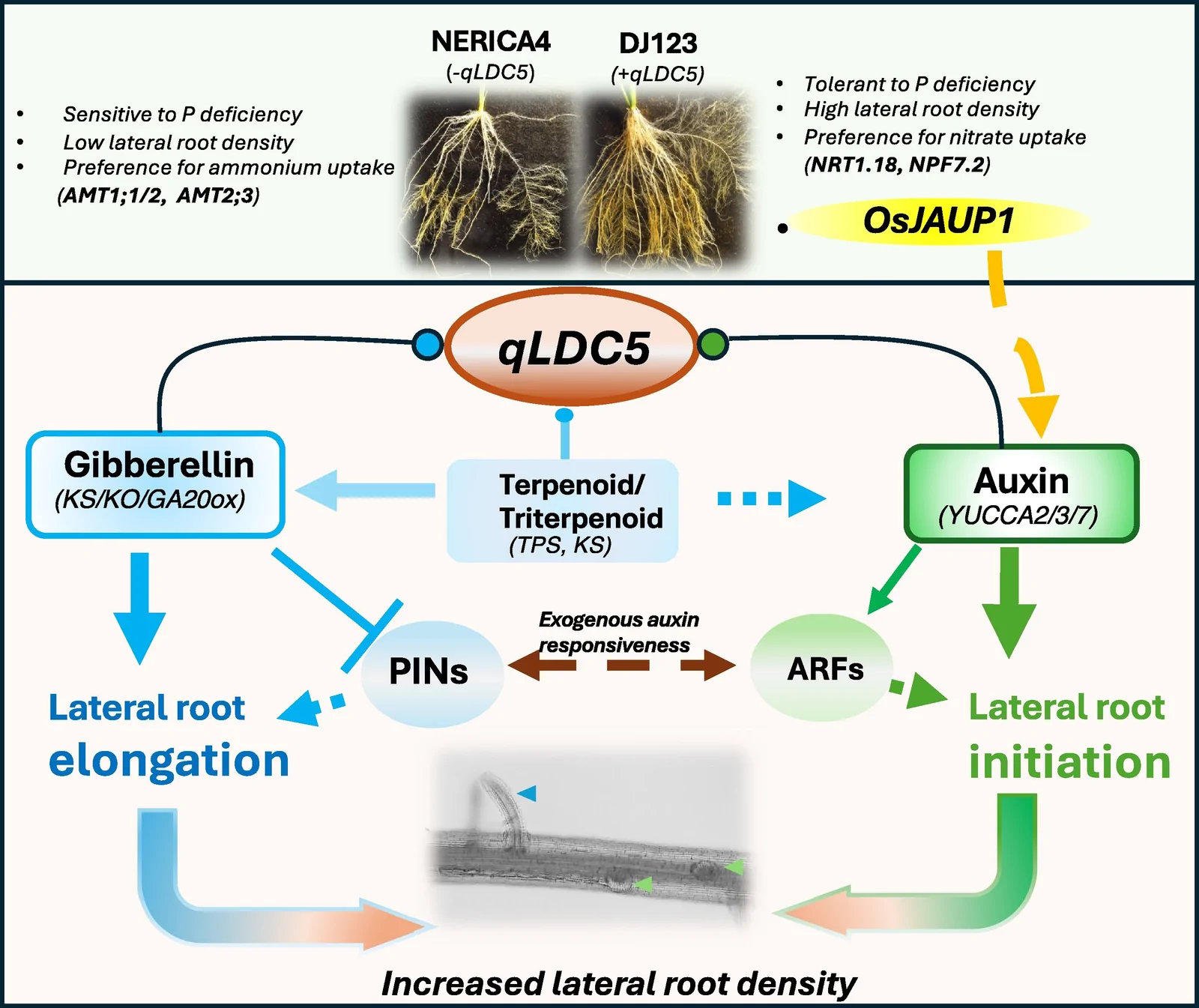
Proposed model for *qLDC5* promoting lateral root density in DJ123, by regulating lateral root initiation and elongation through crosstalk of gibberellin (GA) and auxin. Arrowheads indicate the positive regulation, while the T-bar indicates negative regulation. The dashed arrows indicate feedback and fine-tuning of PIN–ARF crosstalk in response to exogenous auxin in DJ123 and NERICA4. The top panel illustrates genotypic differences in response to phosphorus (P) deficiency and associated lateral root traits. NERICA4 is sensitive to P deficiency, characterized by low lateral root density and a reliance on ammonium-based nitrogen uptake (*AMT1;1/2*, *AMT2;3*) In contrast, DJ123 exhibits tolerance to P deficiency, with high lateral root density and a preference for nitrate-N uptake (*NRT1.18*, *NPF7.2*). A preference for nitrate over ammonium uptake had early been detected in DJ123 (Ueda and Wissuwa, 2022) and has been attributed to enhanced P acquisition efficiency as a result of P solubilization in acidic soil due to increases of rhizosphere pH (Kuppe *et al.*, 2022). Additionally, the higher expression of Jasmonate Upregulated Protein 1 (OsJAUP1) in DJ123 supports a role for jasmonate–auxin interactions in regulating lateral root development. The lower panel shows *qLDC5* increases lateral root density by coordinating gibberellin, auxin, and terpene pathways, thereby regulating both lateral root initiation and elongation. In DJ123, the terpene biosynthesis genes *TPS13* and *TPS19* are up-regulated, providing precursors for gibberellin biosynthesis and potentially influencing auxin signaling indirectly. These terpenes feed into the GA biosynthetic pathway, including *KS3*, *KS4*, *KO4*, and *GA20ox4/5/6/7/9*, which promotes lateral root elongation, partly by modulating PIN-dependent auxin redistribution (*PIN1D/2/3B/5a/10A*), with PIN genes being suppressed. At the same time, the high expression of *YUCCA2*, *YUCCA3*, and *YUCCA7* in DJ123 increases auxin biosynthesis, leading to activation under 100 nM indole-3-acetic acid treatment of *ARF14/15* in DJ123 and *ARF16* in NERICA4, which positively regulate lateral root primordia initiation.

We propose a simple hypothetical model (Fig. 10) to explain the increased lateral root density conferred by the DJ123 allele at *qLDC5*. In DJ123, the terpene biosynthesis genes *TPS13* and *TPS19* are up-regulated, providing precursors for gibberellin biosynthesis and potentially influencing auxin signaling indirectly. These terpenes feed into the GA biosynthetic pathway, including *KS3*, *KS4*, *KO4*, and *GA20ox4/5/6/7/9*, which promotes lateral root elongation, partly by modulating PIN-dependent auxin redistribution (*PIN1D/2/3B/5a/10A*), with PIN genes being suppressed (Fig. 9). At the same time, the high expression of *YUCCA2*, *YUCCA3*, and *YUCCA7* in DJ123 increases auxin biosynthesis, leading under 100 nM IAA treatment to activation of ARF14/15 in DJ123 and ARF16 in NERICA4, which positively regulate lateral root primordia initiation. Overall, the modulation of terpenoid and triterpenoid metabolism, together with coordinated gibberellin–auxin crosstalk, results in the high lateral root density observed in DJ123.

## Conclusion

Using near-isogenic lines, we confirmed that *qLDC5* increases primary root branching, as lines carrying the positive *qLDC5* allele exhibited higher lateral root density, particularly of L-type roots. These results demonstrate that *qLDC5* influences not only crown root branching but also early lateral root development along the primary root, affecting both primordia initiation and subsequent elongation. Our results also highlight that auxin-related pathways regulate *qLDC5*-associated lateral root density: elevated *YUCCA* expression in DJ123 likely enhances local auxin accumulation, while increased *OsARF15* expression may strengthen downstream auxin signaling. Further fine-mapping is underway to determine the gene(s) that directly underlie the *qLDC5* locus.

## Supplementary Material

erag232_Supplementary_Data

## Data Availability

The sequence files supporting the findings of this study are available in the NCBI Short Read Archive (SRA) under the accession ID PRJNA1417494.

## Supplemental data

The following supplementary data are available at *JXB* online.

Fig. S1. Ratio of L-type to S-type lateral root primordia in primary root tips of DJ123, NERICA4, and NDJ188 with or without exogenous IAA, assessed across developmental zones based on primordium size.

Fig. S2. Heat map of 19 selected differentially expressed genes (DEGs) induced by IAA in DJ123 and NDJ188 but not NERICA4, associated with root development regulatory networks (normalized log counts per million).

Fig. S3. Heat map of auxin-responsive and phosphorus-related genes within the *qLDC5* region, highlighting genotype-specific expression patterns, particularly in DJ123 (normalized log counts per million).

Table S1. List of plant materials used in the study.

Table S2. List of 3441 differentially expressed genes (DEGs) common to DJ123 and NDJ188 relative to NERICA4, with expression of *OsJAUP1* and *OsGA2ox5* highlighted.
