# Supplementary material for: Quantitative trait locus qLDC5 regulates primary root branching in an auxin-dependent manner
Source: J Exp Bot. 2026 May 27;77(17):5639–50. doi: 10.1093/jxb/erag232 (PMC13577768; doi:10.1093/jxb/erag232)
Supplement: Supplementary file 1 [file erag232_supplementary_data.zip › erag232_Fig S1-S3f.pdf]

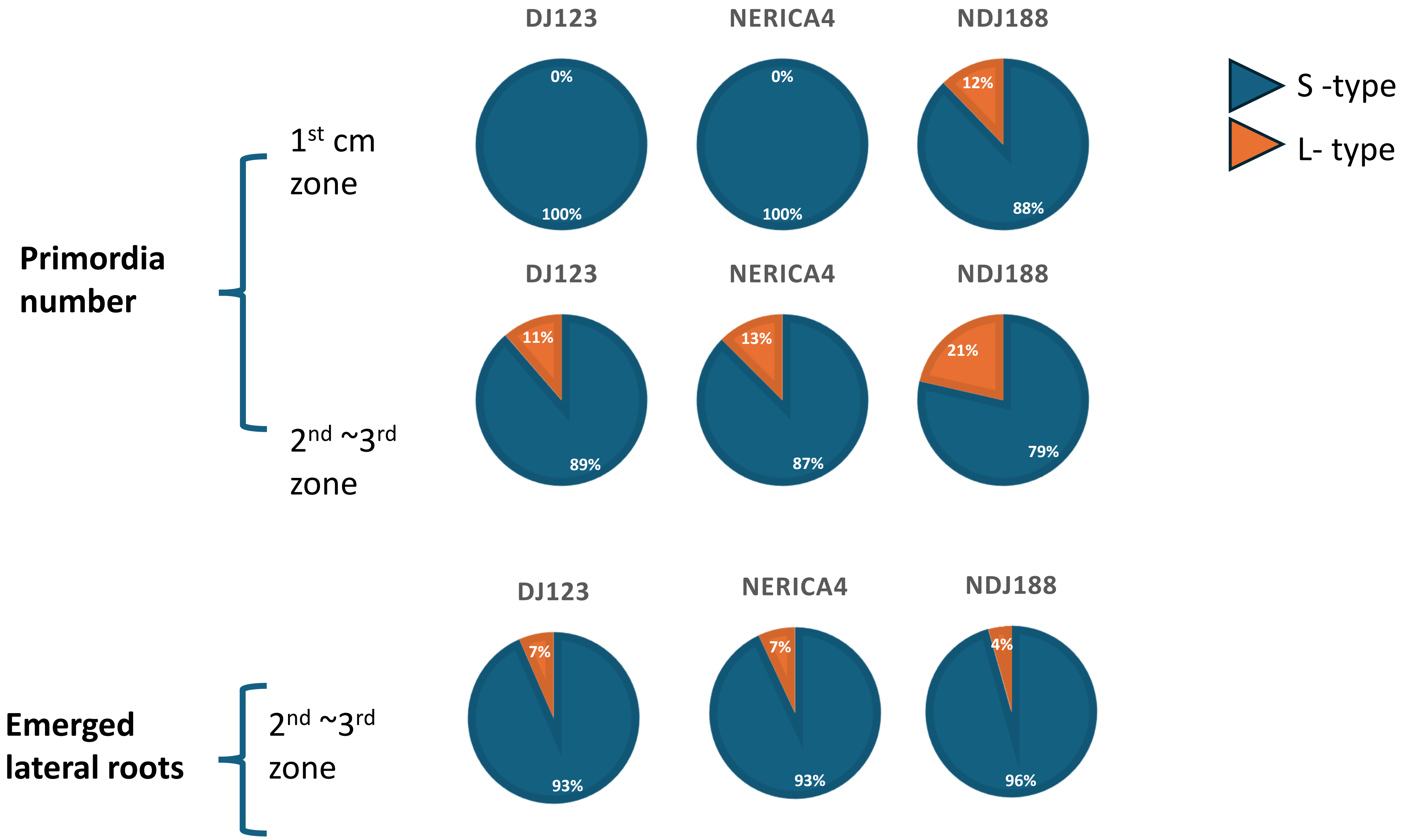

Supplementary Fig. S1. Ratio of L-type to S-type lateral root primordia in primary root tips of DJ123, NERICA4, and NDJ188 with or without exogenous IAA, assessed across developmental zones based on primordium size. Seedlings were grown either without exogenous IAA or with 100 nM IAA applied at 3 days after germination (DAG). Sampling was done on the fi5th day after IAA application, and primordia were counted across different root developmental zones as described in Figure 4A.

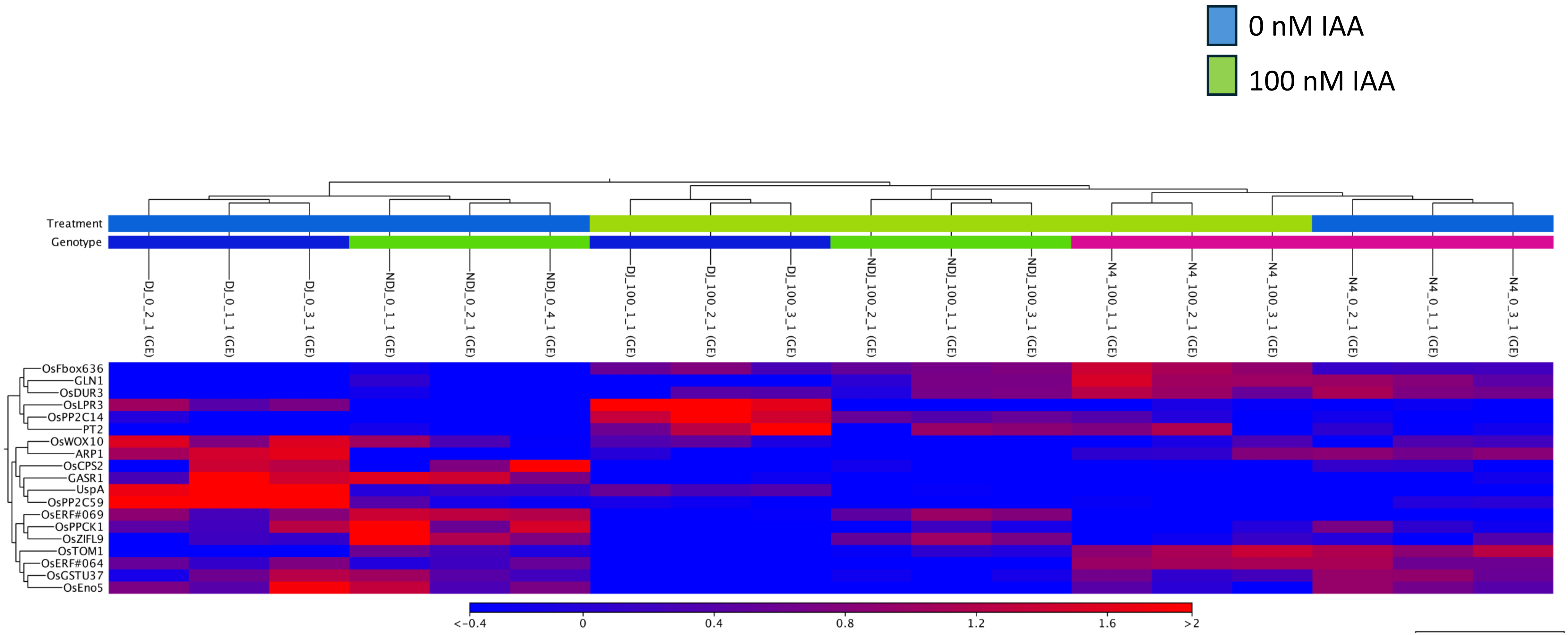

Supplementary Fig. S2. Heat map of 19 selected differentially expressed genes (DEGs) induced by IAA in DJ123 and NDJ188 but not NERICA4, associated with root development regulatory networks. The 19 DEGs were selected based on a previously reported putative role in regulatory networks controlling root development, including hormone signaling, nutrient sensing, metabolic support, and stress adaptation. Each column corresponds to one sample, and each row to a gene. The samples are clustered into three groups according to three genotype DJ123, NDJ188 and NERICA4. The red and blue colors reflect the expression level. The expression values are normalized log CPM values. *CPM: Counts per million.

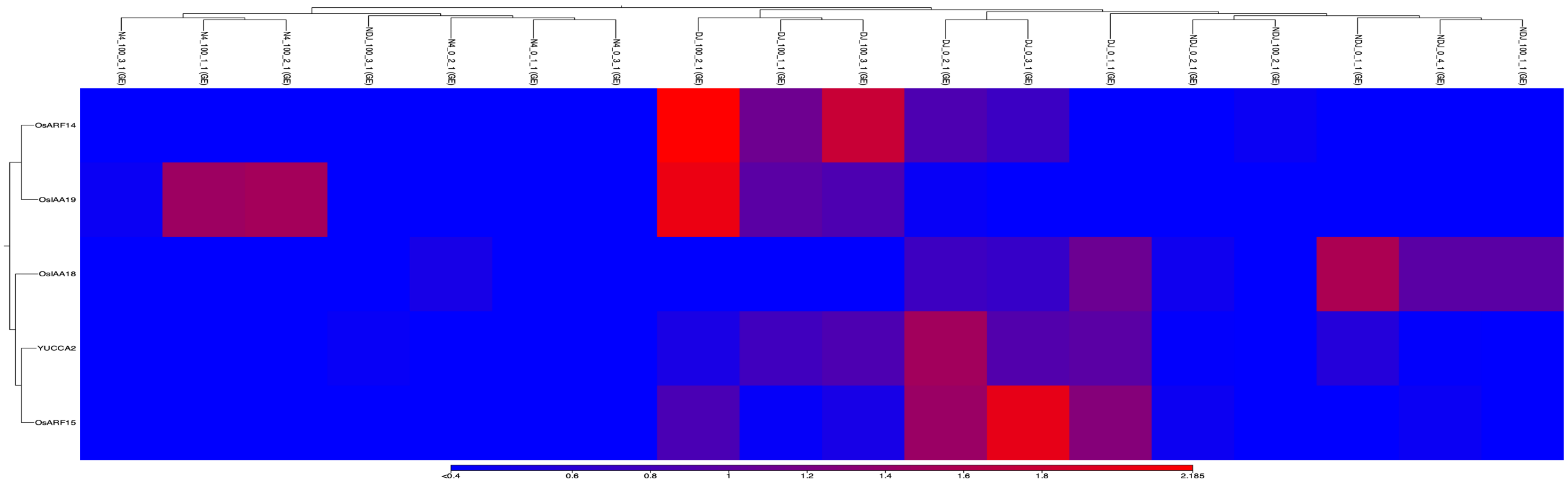

Supplementary Fig. S3. Heat map of auxin-responsive and phosphorus-related genes within the *qLDC5* region, highlighting genotype-specific expression patterns, particularly in DJ123.

In the *qLDC5* interval, genes strongly expressed in DJ123 include *OsIAA18* (auxin signaling), *YUCCA2* (auxin biosynthesis), and *OsARF15* (Auxin Response Factor). Each column corresponds to one sample, each row corresponds to a gene and the red and blue colors reflect the expression level. The expression values are normalized log CPM values. *CPM: Counts per million.
